# Assessing the implementation of performance management of health care workers in Uganda

**DOI:** 10.1186/1472-6963-13-355

**Published:** 2013-09-17

**Authors:** George William Lutwama, Janetta Hendrika Roos, Bethabile Lovely Dolamo

**Affiliations:** 1 Department of Health Studies, University of South Africa, P.O. Box 392, Unisa 0003, South Africa

**Keywords:** Health care workers, Performance management, Performance review, Performance measurement, Performance improvement

## Abstract

**Background:**

The performance management concept is relatively new to the Ugandan health sector. Uganda has been implementing health sector reforms for nearly two decades. The reforms included the introduction of the results-oriented management in the public sector and the decentralisation of the management of health care workers from central to local governments. This study examined the implementation of performance management of health care workers in order to propose strategies for improvement.

**Methods:**

The study was a descriptive survey carried out in the Kumi, Mbale, Sironko and Tororo districts and utilising mixed research methodology. A self-administered questionnaire was used to collect quantitative data from the health care workers. A semi-structured interview guide was used to collect qualitative data from the health service managers. The sample for the quantitative method was selected using stratified random sampling. Purposive sampling was used to select health service managers. Quantitative data were analysed using Statistical Package for Social Sciences (version 18.0). Qualitative data were categorised according to the themes and analysed manually.

**Results:**

The findings show that to some extent performance management is implemented in the health sector; however, there were loopholes in its implementation. There were inadequacies in setting performance targets and performance management planning was hardly done. Although many health care workers had job descriptions, the performance indicators and standards were not clearly defined and known to all workers and managers. Additionally the schedules for performance assessments were not always adhered to. There were limited prospects for career progression, inadequate performance feedback and poor rewarding mechanisms.

**Conclusions:**

Performance management of health care workers is inadequately done in the districts. Performance management is a key component of attempts to improve health sector outcomes. As a result of this study, suggestions to enhance health sector performance management in the districts have been put forward. The authors are optimistic that if these suggestions are implemented, the performance of health care workers is likely to improve.

## Background

Performance management is a vital component of human resource management that ensures the effective use of scarce resources [1]. Performance management is a continuous process of identifying, measuring and developing the performance of individuals or teams and aligning that performance to the strategic goals of the organization [2-4]. Performance management has three main functions which are classified as strategic, administrative and developmental. The strategic function links the workers’ performance to the overall organizational strategy. Administratively, performance management provides valuable information to help the managers make important decisions such as salary increments, promotions, recognition and rewards. The developmental function of performance management is realised through the provision of information on the strengths and weaknesses of health care workers [3,4]. Performance management involves six main steps, which include having knowledge of the job and organization mission, performance planning, performance execution, performance assessment and performance review, as well as performance renewal and re-contracting [3,5].

The concept of performance management is relatively new in the Ugandan health sector and lacks documented proof of practice. Uganda has implemented health sector reforms for nearly two decades to improve access and strengthen health care systems. Among the reforms implemented is the decentralisation of responsibility for delivery of health services and management of health care workers from the central Ministry of Health (MoH) to the local governments. The other reforms included the introduction and later the abolition of service fees, introduction of the Uganda National Minimum Health Care Package (UNMHCP) and the restructuring of MoH [6]. While some components of the health sector reforms have shown favourable results such as the increase in immunization coverage, little emphasis was put on the management of health care workers [7,8]. Human resource management challenges have been reported in Uganda. Among these challenges is weak performance management of health care workers. The value for money audit for the health sector programmes that was carried out in Uganda in the year 2006 revealed a number of weaknesses in performance management of health care workers in the districts. The audit revealed significant staffing gaps with many of the 112 districts in Uganda failing to attract and retain qualified health care workers. In addition, the districts did not have clear policies on staff training, transfers and rotation. There was irregular and inadequate support supervision, and health staff appraisals were occasional. Furthermore, many district and health facility managers were not utilising the existing health management information systems (HMIS) to report performance [9].

Performance management challenges of health care workers are not only unique to Uganda. For example, in Mali the performance management of health care workers is almost non-existent, evidenced by lack of job descriptions, poor training needs analysis, subjective support supervision and appraisal systems [10]. In Uganda, despite the health sector reforms, the health services are not yet up to the required standards. The inadequate performance management might be one of the contributing factors to the deterioration or stagnation of some of the important health indicators under the third health sector strategic plan [11].

The health care system in Uganda consists of national and regional referral hospitals and the district health system. The district health care system comprises the district general hospitals, health centres (HC) levels II, III and IV, and the village health teams (VHTs). These levels are closely linked to the political and administrative structures of local government. For example, HC II serves the parish or ward, HC III serves the sub-county and HC IV serves the county or parliamentary constituency [12,13].

Up to the time of this study performance management of health care workers in Uganda was not adequately documented. The objective of this study was to investigate the performance management of health care workers in Uganda, specifically to identify the current practices used to manage the performance of health care workers and to propose strategies for improvement. The findings of this study will provide policy makers with a framework to guide planning and management of health care workers not only in Uganda but also in other developing nations.

## Methods

The study was a descriptive survey using mixed research methodologies and carried out in the districts of Kumi, Mbale, Sironko and Tororo. Mixed research methodology is an approach where both quantitative and qualitative methods are used at the same time or in a series of studies [14]. In this study, quantitative methods were used to collect data from health care workers and qualitative methods from health service managers. Both types of data were collected concurrently and with equal priority between April and June 2010. Triangulation was done during data collection, analysis, interpretation and discussion of the results.

### Quantitative method

The study population comprised health care workers (doctors, clinical officers and professional nurses) from the health facilities in the selected districts. In order to avoid bias and ensure representativeness, stratified random sampling was used to select the respondents [15,16]. Stratification was done by district, profession and type of health facility where respondents worked. Using simple random sampling, health care workers were selected from each stratum based on the lists obtained from the district health offices. A total of 331 respondents were selected to participate in the study. This represented slightly over forty per cent of the target population.

The data were collected using self-administered questionnaires. The current practices used to measure performance of health care workers were assessed using eight questions. Six questions, based on a five-point Likert scale, required the respondents to indicate their level of agreement with the statements. These six questions elicited information regarding performance management under these themes: setting performance standards (8 items), performance measurement (9 items), performance reporting (6 items), performance improvement (10 items), rewarding system (6 items), and staff training and development (9 items). The remaining two questions, one concerning “how performance is reviewed”, required respondents to circle only one appropriate answer and the other on “how performance information is utilised” had six items which required yes or no answers.

Prior to data collection, the questionnaire was pre-tested. The pre-testing of the questionnaire was done by issuing the questionnaire to seven experts (human resource managers, public health specialists, a statistician and promoters) as well as 12 experienced health care workers. Minor modifications in the wording of items on the questionnaire were necessary. The first author delivered the questionnaires with the help of trained research assistants. The reasons for undertaking the research were explained to the respondents in a letter. Written consent was obtained from all the respondents. The questionnaires were left with the respondents to complete. After two weeks the completed questionnaires were collected from the respondents, checked for completeness, coded, entered in the computer and analysed using the Statistical Package for Social Sciences (version 18.0). Statistical calculations were done using descriptive statistics. The categories “strongly disagree” and “disagree” as well as the categories “strongly agree” and “agree” were combined for data presentation and interpretation to mean “disagree” and “agree” respectively. The results are presented in the tables.

Stratified random sampling, adequate sample size and correct composition of the sample were some of the measures the researchers put in place to ensure external validity. Another measure was to collect data from all the levels of health facilities, including hospitals and health centres. Measures to ensure internal validity were the construction of the questionnaire based on the objectives of the study, an extensive literature review and the voluntary participation of the health care workers. The internal consistency of the questionnaire was established by means of Cronbach’s alpha (α). The overall alpha (α) was 0.86 which is adequate [17].

### Qualitative method

The study population comprised health service managers at district, hospital and health sub-district levels. The health service managers were purposively selected for inclusion. Data from the health service managers were collected using a semi-structured interview guide through face-to-face interviews. A total of 21 health service managers were interviewed. The interviews were conducted in English. Before the interview commenced, the interviewers explained the purpose and the procedure of the interview and obtained written informed consent and permission to use a tape recorder. The participants were assured of confidentiality for all information they provided. They were also informed that they would be free to pull out of the interview at any point. The data collected were transcribed verbatim, coded and analysed manually. Analysis involved creating categories, refining them and grouping them into themes and sub-themes before presentation, interpretation and discussion.

The rigour and validity of the data were ensured by audio recording, taking comprehensive notes, and verbatim transcription of the interviews. The rigour was also enhanced through methodological triangulation and involving the statistician and the supervisors of the study to cross-check the coding to ensure consistency between the themes, sub-themes and categories.

### Ethical approval

The research protocol was approved by the Research and Ethics Committee of the Department of Health Studies of the University of South Africa, Uganda National Council for Science and Technology and Institutional Review Board of Mbale regional referral hospital. In the study area, consent for data collection was obtained from the relevant district authorities. All respondents and participants signed an informed consent form and were assured of confidentiality and anonymity. Their right to withdraw from the study or to answer specific questions was explained to them. The privacy of the respondents and participants was respected at all times.

## Results

Out of 331 respondents selected for the study, 276 completed and returned the questionnaires. Therefore a response rate of 83.4% was achieved.

### Characteristics of respondents involved in the quantitative method

The respondents were mostly females (63.8%) and aged between 30 to 49 years (62.4%). A total of 74.6% belonged to the professional nurse and midwife cadres, 18.5% were clinical officers and 6.9% were medical doctors. The respondents held mainly diplomas (53.6%). Many respondents (73.5%) had professional experience of more than five years. More than half of the respondents (56.9%) were working in health centres (levels II, III, IV), while 43.1% were hospital based. A total of 73.5% had worked in their respective health facilities for at least six or more years. Nearly sixty per cent of respondents (59.8%) were working in rural areas, while 40.2% were based in urban areas. Slightly over thirty-eight per cent (38.4%) were working in Mbale district, 23.6% in Tororo district, 19.9% in Kumi district and 18.1% in Sironko district (see Table 1).

**Table 1:** Description of the respondents involved in the quantitative method of the study (n=276)

**Demographic characteristics**	**Number**	**Percentage (%)**
**Sex**		
Female	176	63.8
Male	100	36.2
Age (in years)		
20 to 29	50	18.1
30 to 39	115	41.7
40 to 49	57	20.7
50 and above	54	19.6
**Profession**		
Enrolled nurse/midwife	95	34.4
Registered nurse/midwife	111	40.2
Clinical officer	51	18.5
Medical doctor	19	6.9
**Level of education**		
Certificate	94	34.1
Diploma	148	53.6
Bachelor’s degree	23	8.3
Postgraduate certificate/diploma	5	1.8
Master’s degree and above	6	2.1
**Years of experience**		
0 to 5	73	26.4
6 to 10	68	24.6
11 to15	43	15.6
16 to 20	26	9.4
21 and above	66	23.9
**Level of health facility**		
Health centre II	21	7.6
Health centre III	80	29.0
Health centre IV	56	20.3
Hospital	119	43.1
**District**		
Mbale	106	38.4
Tororo	65	23.6
Kumi	55	19.9
Sironko	50	18.1

### Characteristics of participants involved in the qualitative method

The majority of the participants (health service managers) were male (76.2%). A high percentage (66.7%) was aged between 30 and 49 years. Of the health service managers, 33.3% were from Sironko district, followed by Kumi district (28.6%). Both Mbale and Tororo districts were represented by 19.1% managers for each. Of the health service managers, 80.9% had worked in their respective districts for six or more years. Of the managers 76.2% revealed that they had some form of training in general management or human resource management.

### Performance management

This section presents the analysis pertaining to the implementation of performance management in the health sector. The dimensions reviewed include setting performance standards, performance reviews, performance reporting, performance improvement, rewarding system, staff training and development and use of performance review information.

### Setting performance standards

The majority of health care workers (70.3%) indicated that they had clear job descriptions and that targets to be achieved were set. A total of 9.8% health care workers mentioned that no target setting for activities takes place. About 69.9% of the health care workers stated that they were familiar with their organizations’ mission, had clear performance standards and relevant performance indicators.

Slightly over sixty per cent (60.1%) of the health care workers affirmed that all organizational stakeholders participated in setting performance standards and that those performance standards, indicators and targets are communicated to all departments. A similar proportion (60.1%) of the health care workers mentioned that their organizations regularly report performance standards and indicators to external stakeholders. However, 29.7% of health care workers disagreed that all stakeholders were involved in setting performance standards and also disagreed that the organizations regularly report performance indicators to external stakeholders, while 10.1% were undecided (see Table 2).

**Table 2:** Items concerning setting performance standards (n=276)

**Item description**	**Disagree**		**Undecided**		**Agree**		**Total**	
	**n**	**%**	**n**	**%**	**n**	**%**	**n**	**%**
All health care workers are familiar with the organization’s mission towards clients	28	10.1	55	19.9	193	69.9	276	99.9
I have a clear job description	82	29.7	0	0.0	194	70.3	276	100.0
The performance standards are clear	55	19.9	28	10.1	193	69.9	276	99.9
There are appropriate performance indicators to assess the health care worker’s performance	83	30.1	0	0.0	193	69.9	276	100.0
Targets are set for activities to be achieved in a given period	27	9.8	55	19.9	194	70.3	276	100.0
The performance standards, indicators, and targets are communicated to all departments to ensure that health care workers understand them	55	19.9	55	19.9	166	60.1	276	99.9
This organization regularly reports the performance of standards, indicators and targets to the external stakeholders	82	29.7	28	10.1	166	60.1	276	99.9
All the stakeholders in this organization participate in setting performance standards	82	29.7	28	10.1	166	60.1	276	99.9

The data from the health service managers revealed that setting performance standards and targets posed a huge challenge in the districts. Most of the managers acknowledge that clear performance appraisal guidelines exist, but performance agreements were inadequately done. Some managers indicated that the supervisors and their subordinates hardly sat together to set targets and agree on the expected standards, as articulated in this quotation:

*… [T]he biggest problem is actually setting targets. In fact what our staff put on their appraisal forms are not targets but are just statements…* (Health Service Manager, Mbale district, 2010–04).

### Performance reviews

Nearly half (49.6%) of the health care workers indicated that there were formal and regular performance reviews, 30.4% indicated that there were informal but regular reviews and 19.9% mentioned the existence of some informal but unscheduled performance reviews.

All health service managers reported that there is some form of performance review in their districts. Most health service managers, however, mentioned that pre-appraisal planning meetings are hardly done, as indicated in the following quotation:

*We hardly plan for this … I would say it is just a routine that you fill these appraisal forms, you don’t even sit to discuss with the person concerned, you also just tick and evaluate without making realistic comments…* (Health Service Manager, Kumi district, 2010–05).

A performance meeting helps supervisors and employees to establish employee’s job duties, responsibilities and priorities. The following quotation however shows that it does not take place on a regular basis:

*… [Y]es we have these meetings on paper but not in practice …* (Health Service Manager, Sironko district, 2010–05).

### Performance measurements

The majority of health care workers (80.1%) indicated that their performance was evaluated based on their job descriptions and that the objectives to be achieved were known by the staff concerned. Most health care workers (69.9%) agreed that their districts clearly define how individual performance is measured and ensure that the expected performance standards are clearly understood by all. Many health care workers (69.9%) indicated that their organization has a system for collecting and tracking staff performance data and 30.1% disagreed (see Table 3).

**Table 3:** Items concerning performance measurement (n=276)

**Item description**	**Disagree**		**Undecided**		**Agree**		**Total**	
	**n**	**%**	**n**	**%**	**n**	**%**	**n**	**%**
Objectives to be achieved are known by individuals to be assessed	55	19.9	0	0.0	221	80.1	276	100.0
The performance standards expected from the staff are clear and understood by all	83	30.1	0	0.0	193	69.9	276	100.0
The district clearly defines how to measure individual activity performance	83	30.1	0	0.0	193	69.9	276	100.0
This organization has a system for collecting and tracking staff performance data	83	30.1	0	0.0	193	69.9	276	100.0
The organization measures most of the established individual performance standards and targets	83	30.1	0	0.0	193	69.9	276	100.0
Individual health care worker’s performance is measured regularly	55	19.9	55	19.9	166	60.1	276	99.9
I am fully aware of the process used to measure my performance	56	20.3	54	19.6	112	60.1	276	100.0
My performance is evaluated based on my job description	28	10.1	27	9.8	221	80.1	276	100.0
My performance is fairly measured	28	10.1	55	19.9	193	69.9	276	99.9

Similarly, 69.9% of the workers indicated that their performance was fairly measured; 19.9% were undecided and 10.1% disagreed. Approximately 60.1% of health care workers agreed that their performance was measured regularly and they are fully aware of the processes involved.

Performance measurement was not done as regularly and as frequently as it was supposed to be done. One of the managers clearly stated:

*… [P]erformance measurement is done here … but to me this is really ad hoc …* (Health Service Manager, Mbale district, 2010–04).

Some health service managers mentioned that there were no clear tools for measuring performance, while others also reported a lack of clear indicators for measuring performance of health care workers. These might be reasons why performance measurement was done in such a haphazard way.

### Performance reporting

The majority of the health care workers (80.1%) agreed that their organizations had a specific system that regularly reports performance of health care workers. Most health care workers (80.4%) indicated that they were granted opportunities to make comments about their performance results and 69.9% of them indicated that their organizations documented progress related to the performance standards and targets. Furthermore, 60.5% of health care workers agreed that performance data were analysed and reviewed according to the set standards, indicators and targets.

A total of 59.8% health care workers reported receiving regular, constructive feedback from performance appraisals. About 40.6% of the health care workers agreed that health care workers’ performance was reported to the external stakeholders (clients, community leaders, suppliers of inputs, leaders from non-profit, private and public sectors); 30.1% disagreed and 29.3% were undecided (see Table 4).

**Table 4:** Items concerning performance reporting (n=276)

**Item description**	**Disagree**		**Undecided**		**Agree**		**Total**	
	**n**	**%**	**n**	**%**	**n**	**%**	**n**	**%**
This organization documents the progress related to performance standards and targets	28	10.1	55	19.9	193	69.9	276	99.9
This organization has a specific system that regularly reports the performance of health care workers	55	19.9	0	0.0	221	80.1	276	100.0
Constructive feedback on performance appraisal is provided on a regular basis	84	30.4	27	9.8	165	59.8	276	100.0
This organization always reports the health care workers’ performance information to the external stakeholders	83	30.1	81	29.3	112	40.6	276	100.0
The health care workers’ performance data are analysed and reviewed according to the set performance standards, indicators and targets	55	19.9	54	19.6	167	60.5	276	100.0
The health care workers are given opportunity to make comments on the results of their performance	0	0.0	54	19.6	222	80.4	276	100.0

The managers expressed the need for supervisors to provide the health care workers with the feedback every time an appraisal is done. They declared that face-to-face discussions of appraisal results were the appropriate way for showing the health care workers their strengths and weaknesses:

… *I really encourage all the supervisors to have face-to-face meetings and discussions with their supervisees concerning their performance. Is it done the right way? I am not certain …* (Health Service Manager, Tororo district, 2010–05).

Some managers stated that though they measure performance, they do not always discuss it with the employees because they are afraid to create conflict with their subordinates, especially if their performance was not good.

### Performance improvement

The majority of the health care workers (89.9%) indicated that they always have access to their supervisors whenever they need support. Of the health care workers, 69.9% indicated that their supervisors encouraged them to use different ways to improve performance and that the workers are involved in performance improvement decisions. Many health care workers (60.1%) agreed that the analysis of their training needs was based on the performance appraisal results, while 30.1% disagreed and 9.8% were undecided. A little over a half (50.4%) of the health care workers indicated that there were procedures to gather employees’ suggestions for performance improvement; 39.9% disagreed and 9.8% were undecided. Another 49.6% of the health care workers agreed that performance data is used to set priorities for personal development and timely actions are taken when performance falls below acceptable levels (see Table 5).

**Table 5:** Items concerning performance improvement (n=276)

**Item description**	**Disagree**		**Undecided**		**Agree**		**Total**	
	**n**	**%**	**n**	**%**	**n**	**%**	**n**	**%**
Timely action is taken when performance falls below the acceptable levels	84	30.4	55	19.9	137	49.6	276	99.9
The performance reports are effectively used for decision making	57	20.7	110	39.9	109	39.5	276	100.1
The health care workers’ performance information is used to set priorities for personal development	57	20.7	82	29.7	137	49.6	276	100.0
The staff is involved in decisions about performance improvement	56	20.3	27	9.8	193	69.9	276	100.0
The organization has specific processes to manage changes in policies, programmes or infrastructure	28	10.1	136	49.3	112	40.6	276	100.0
My supervisors encourage me to use different ways to improve my performance	55	19.9	28	10.1	193	69.9	276	99.9
Rewards and sanctions are based on performance results	85	30.8	54	19.6	137	49.6	276	100.1
The analysis of employees’ training needs is based on the performance appraisal reports	83	30.1	27	9.8	166	60.1	276	100.0
There are procedures to collect suggestions for performance improvement from the employees	110	39.9	27	9.8	139	50.4	276	100.0
I always have access to my supervisors when I need support	28	10.1	0	0.0	166	89.9	276	99.9

Similarly, 49.6% of the health care workers agreed that rewards and sanctions were based on performance results, 30.8% disagreed and 19.6% were undecided. Some health care workers (49.3%) were doubtful about the existence of specific processes to manage changes in policies, programmes and infrastructure for performance, 40.6% agreed and 10.1% disagreed. Slightly less that forty per cent (39.5%) of health care workers agreed that the performance appraisal reports were used for decision making, 39.9% were undecided and 20.7% disagreed.

All the health service managers concurred that the performance management should be a continuous and interactive process.

… *[T]he supervisors need to sit and discuss with their subordinates to agree on the objectives and set realistic targets …* (Health Service Manager, Mbale district, 2010–05).

Some managers also suggested that the appraisal process should be a two-way one. In addition, some managers proposed that the appraisal tool be made available at all times. The process should be clearly explained to the health care workers and the indicators for measuring individual performance should be available and known to all the stakeholders. Managers recommended that performance feedback should be given regularly to health care workers. They also believed that in order to improve performance appraisal, supervisors and their subordinates need to be enlightened about performance management and its importance.

### Reward system

Most health care workers (80.1%) were dissatisfied with their fringe benefits and 70.3% indicated that their salaries are aligned to their job responsibilities.

A total of 60.5% health care workers disagreed that all workers know their fringe benefits; 29.7% agreed and 9.8% were undecided. Of the health care workers, 50.4% indicated that their organizations do not offer sufficient opportunities for promotions, while 49.6% agreed that their organizations offered promotional opportunities. Another 39.5% of health care workers were uncertain on whether hard work was acknowledged and rewarded; 30.1% agreed and 30.4% disagreed. Some health care workers (39.5%) agreed that they were paid according to their experience, 30.4% disagreed and 30.1% were undecided (see Table 6).

**Table 6:** Items concerning the reward system (n=276)

**Item description**	**Disagree**		**Undecided**		**Agree**		**Total**	
	**n**	**%**	**n**	**%**	**n**	**%**	**n**	**%**
I am paid according to my experience	84	30.4	83	30.1	109	39.5	276	100.0
My salary is according to my job responsibilities	82	29.7	0	0.0	194	70.3	276	100.0
Hard work is acknowledged and rewarded accordingly	84	30.4	109	39.5	83	30.1	276	100.0
All health care workers know their fringe benefits	167	60.5	27	9.8	82	29.7	276	100.0
I am satisfied with the fringe benefits I get from my organization	221	80.1	0	0.0	55	19.9	276	100.0
I feel my organization offers sufficient opportunities for promotion	139	50.4	0	0.0	137	49.6	276	100.0

When the health service managers were asked how they recognised performance of health care workers in the districts, they all indicated issuing certificates, end-of-year parties, material gifts and words of praise. One manager remarked:

*I can confidently say that in our district we have some rewards or recognition we give to our workers for good performance but …* (Health Service Manager, Tororo district, 2010–05).

Managers suggested that good performance of health care workers should be rewarded in order to motivate them. The discussions revealed that managers believed that when health care workers are motivated they are extremely likely to be enthusiastic about performance appraisal. The rewards that were proposed include recommendation for training, promotion in service, giving more responsibility and issuing letters of recognition, as well as certificates of achievement or recognition.

### Staff training and development

Most of the health care workers (90.2%) indicated that they had received the training required to succeed in their positions and 79.7% also agreed that appropriate training was conducted to ensure that they carried out their duties well. Some health care workers (69.9%) agreed that they have participated in identifying their career development needs, as indicated in Table 7.

**Table 7:** Items concerning staff training and development (n=276)

**Item description**	**Disagree**		**Undecided**		**Agree**		**Total**	
	**n**	**%**	**n**	**%**	**n**	**%**	**n**	**%**
This organization has a staff training and development policy	110	39.9	55	19.9	111	40.2	276	99.9
Opportunities exist for career advancement in this organization	110	39.9	0	0.0	166	60.1	276	100.0
Appropriate training is conducted to ensure that health care workers carry out their duties well	56	20.3	0	0.0	220	79.7	276	100.0
Job specific refresher courses are provided on a regular basis	137	49.6	27	9.8	112	40.6	276	100.0
The in-service training provided is adequate to deal with the existing skills gap	137	49.6	28	10.1	111	40.2	276	99.9
Health care workers who are less competent are provided with the necessary support to improve their knowledge and skills	83	30.1	54	19.6	139	50.4	276	100.1
Health care workers participate in identifying their career development needs	28	10.1	55	19.9	193	69.9	276	99.9
In the last 6 months my supervisors discussed my career development prospects with me	166	60.1	0	0.0	110	39.9	276	100.0
I have received the training required to succeed in my position	27	9.8	0	0.0	249	90.2	276	100.0

Of the health care workers, 60.1% agreed that opportunities existed for career advancement in their respective organizations and 39.9% disagreed. Most respondents (60.1%) disagreed on discussing their career development prospects with their supervisors during the past six months and 39.9% agreed. Slightly over half of the health care workers (50.4%) indicated that those workers who were less competent were provided with the necessary support to improve their knowledge and skills, 30.1% disagreed and 19.6% were undecided. Some health care workers (49.6%) disagreed that the in-service training was adequate to deal with the existing skills gaps and that there were regular job-specific refresher courses, 40.6% thought there were regular job-specific refresher courses and 9.8% were undecided. Some (40.2%) health care workers agreed that there was a training and development policy, 39.9% disagreed and 19.9% were undecided (see Table 7).

All the health service managers mentioned that they had some short training courses in their districts for all cadres to equip them with up-to-date skills for performance enhancement. This is what one manager had to say:

*… [T]hen the refresher courses to new staff that are recruited … There are many clinical officers who [are] heading health units but many of them do not know how to fill them …* (Health Service Manager, Sironko district, 2010–04).

All health service managers noted that though the training courses are available, they are not conducted regularly as there is lack of funding.

### Use of performance review information

The majority of the health care workers (90.2%) were of the view that the information from the performance appraisals was utilised in some ways to improve performance.

Of the health care workers, 80.4% indicated that performance review data were used for promotions; 79.7% said data were used for identifying the training needs and 69.9% indicated that data were used for rewarding and rotating staff. Most health care workers (80.1%) did not think that the results of the appraisals are used for demoting staff.

The views of the managers were divided on this issue. Some of the health service managers stated that the results of performance appraisals were not used in their facilities. Most managers indicated that the results were used to identify training needs of the health care workers. One manager regretted, however, that training needs were identified but did not guarantee sponsorship for the candidates. Other managers said they used the results for promotion, support supervision, confirmation and staff re-distribution purposes (See Table 8).

**Table 8:** Use of performance review data (n=276)

**Use of performance review information**	**Yes**		**No**		**Total**	
	**n**	**%**	**n**	**%**	**n**	**%**
Training of staff	220	79.7	56	20.3	276	100
Promotion in service	222	80.4	54	19.6	276	100
Demotions of staff	55	19.9	221	80.1	276	100
Rotation of staff	193	69.9	83	30.1	276	100
Rewards	193	69.9	83	30.1	276	100
Not used at all	27	9.8	249	90.2	276	100

## Discussion

The findings of this study uncovered various methods used to review the performance of health care workers and these included formal and regular reviews, informal but regular reviews and some informal and unscheduled reviews. It is evident that performance reviews were done in the districts. Similar results were also reported in Kenya. However, in Benin there were no performance reviews for health care workers [18]. Performance management tools serve to improve the performance of health care workers and assist in setting, communicating and internalising the organizational goals [18].

The results show that the MoH in Uganda sets some standards for performance management. Performance standards and indicators facilitate accountability, monitoring health care systems, modifying health care workers’ behaviour and informing policy initiatives [19]. This result is almost similar to what was found in Malawi, where the MoH issues performance management standards in the form of checklists [20]. This study has revealed that many health care workers have clear job descriptions. Having clear job descriptions and knowledge of the organization mission are some of the building blocks for performance management. Job descriptions with clearly stated objectives, authority and lines of accountability are mostly associated with improved achievement of work goals [21]. The finding of this study contrasts with what was discovered by another study in Malawi where less than a quarter of the health care workers had written job descriptions, and that the written job descriptions possessed by the remainder did not even match their assigned duties [22].

Most of the managers mentioned that many low-cadre staff members might not be aware of the mission of their organization. In order to have an efficient performance management system, the workers must have knowledge of the organization’s mission, strategic goals and the job [5].

Regarding target setting, there were differences between the responses of health service managers and those of health care workers. Whereas most health care workers indicated that target setting existed, most health service managers reported that pre-appraisal planning and meetings rarely take place. The majority of managers mentioned that even though performance management guidelines were available, target setting was inadequately done; pre-appraisal planning meetings were rarely held. This is a big challenge which the MoH needs to meet if the performance of health care workers is to improve. Performance planning discussions and agreements between the supervisor and the health care worker must deal with performance targets and strategies. Without proper targets and performance agreements in place it is almost impossible to monitor the performance of health care workers. A Malawian study showed almost the same results, whereby neither performance targets nor timelines to allow progress to be measured were instituted [22]. These adversely affect the quality of the performance management process.

The findings of this study have established that there are standards set by the MoH to measure the performance of health care workers. It was, however, noted that not all health care workers knew what performance measurement was all about and what indicators were applied (see Table 4). This finding approximates to findings in Kenya which demonstrated that, although health care workers underwent annual performance assessments, not everybody knew what they comprised [18]. All health care workers should be made aware of performance management and its importance to improving health service delivery as well as their individual capabilities.

Many health service managers mentioned lack of definite tools for measuring performance since even the appraisal forms from the Ministry of Public Service lacked clear indicators for measuring and monitoring performance. In the districts where performance measurement was done, the managers knew that it was supposed to be done regularly; yet they did it on an ad hoc basis, as was the case elsewhere in Africa [20,21]. The Ugandan Ministry of Public Service’s guidelines stipulate that performance appraisal should to be done every six months for the health care workers on probation and annually for permanent employees [23]. In practice this was hardly ever done. It is the responsibility of the health service managers to ensure that performance management standards are disseminated to all the health care workers in the districts. The district health managers should then follow up directly with staff working in the health facilities under their jurisdiction.

The results show that performance assessment data were used mainly for sound motives such as identifying training needs, promotions, staff rotation, determining who should be rewarded and in some cases demotion of staff. In addition, the data results were used for identifying the support supervision needs, confirmation of staff members in public services and for staff redistribution. Studies have demonstrated that human resource management interventions like training, local performance analysis and payment combined with organizational transformation could actually improve health care workers’ performance [24]. It is worth noting that some health care workers and managers pointed out that performance review data were not used at all. If indeed this is true then the health service managers at the respective levels should be held accountable for this setback. It is the responsibility of all stakeholders in the health sectors to ensure that data collected is put to good use. This is the only way health sector performance can be improved.

There were diverse responses from health care workers regarding performance reporting. Most health care workers thought that their supervisors report their performance. Some of them did not receive regular and constructive feedback about their performance. There were some workers who did not believe that health service managers report the health care workers’ performance results to the external stakeholders. These findings are consistent with results from studies in Benin, Kenya, Malawi and Tanzania, where performance reporting posed challenges in the health sectors [18,20,22,25].

From the health service managers’ perspective there were still differences concerning performance reporting, with slightly less than half admitting that they did not report performance. Performance reporting is an important component for performance management, which needs to be implemented at all levels if improvement in the health sector is to be realised. The reporting should be done frequently and regularly. The supervisors should be in a position to provide individual performance feedback every time an assessment has been done. Some researchers have established that employees without good-quality relationships with their supervisors do not have as much access to information that lets them know how well they are meeting performance standards [26]. Previous studies show that sharing information helps health care workers perform better [27,28].

This study has revealed that managers believed in face-to-face discussion of performance assessment results as a way of showing health care workers their strengths and weaknesses. Without a functional feedback mechanism, the health care workers were likely to be unmotivated to participate in the performance management process since they did not see its benefits. Hence, the organizations need to develop a culture of monitoring, evaluation and feedback of health information to improve the performance of health care workers.

The results of this study revealed some deficiencies in the area of performance improvement. The data collected from the performance management system are not adequately used to improve health service delivery. Without proper analysis of the performance assessment results, health service managers cannot implement corrective interventions. In order to ensure an efficient performance management system, health care workers should be given a chance to make suggestions on the areas for improvement. Staff participation and involvement is an important human resource management function. It allows the best use of health care workers’ knowledge, hands-on experience and ideas for improvement [18]. This has an important effect on motivation and performance of health care workers. Additionally, the supervisors should be available to guide health care workers to perform up to the expected standards. Quality health improvement can only be achieved when health care workers clearly know the outcomes they are working towards, as well as what changes can lead to improvements, and how to evaluate their efforts [29].

The findings provide a clear picture of how deficient the reward system is in the districts under study. This is likely to have a negative bearing on the performance of health care workers. Rewards and remuneration systems are believed to influence the behaviours and performance of health care workers [30]. The World Health Organization emphasises that health care workers must be remunerated well for the work they perform [31]. If health care workers are not paid well, they are likely to adopt some coping mechanisms such as absenteeism, migration, creation of ghost workers and referral of patients to the private sector where some health care workers hold part-time jobs [32]. Performance-related payment methods are increasingly being applied in many developed countries and some developing countries such as Rwanda have adopted such systems [28].

It is important to note that many health service managers recognised the significance of rewarding good performance as a way of improving the performance of health care workers, even though there were no formal mechanisms at the moment for rewarding good performance in some districts. This is almost the same to what was found in Mali, where no formal system exists to show appreciation for good performance [10]. The health service managers proposed a number of ways that may be used to recognise performance of health care workers, such as issuing certificates of achievement, offering gifts for outstanding performance, end-of-year parties and words of praise. Performance management should serve to identify who is eligible for pay rises, promotions and placement and accordingly motivate employees in their jobs and give constructive feedback for improvement [3,33]. For poor performance, performance management should identify areas for improvement, and design strategies and mechanisms for improvement. Active involvement of health care workers should be encouraged to identify and implement solutions to improve their performance challenges. Such solutions could be focused training, continuing medical education and regular support supervision [24].

This study shows that district managers attempt to meet the short-term training needs of the health care workers. Some health care workers mentioned that they have opportunities for career development. Training and professional development are important motivation and performance determinants as they nurture the health care workers’ personal objectives. This is likely to help the health care workers cope better with the requirements of their jobs [18]. In-service training is one of the commonly used methods in the health sector to enhance performance of health care workers in the districts. Researchers argue that adequate in-service training is necessary for quality health service delivery in primary health care settings [34]. Policy changes, new clinical guidelines and general management tasks all require effective training of as many health care workers as possible in order to reach the programme objectives. Studies have demonstrated that professional development can influence the health care worker’s motivation as well as performance [27,35]. Therefore, in order to realise significant changes in the quality of services in Uganda, there is a need to adequately meet the training needs of the health care workers. This will improve their motivation and their capacity to manage challenges in the health sector.

## Proposed strategies for improving performance management in the health sector

Based on the findings from this study, we recommend the following strategies for improving performance management in Uganda.

### Understanding the context of performance management in the health sector

The district health service managers must continually scan the environment where the health care workers are operating in order to identify factors that may affect performance management. They also need to prepare and educate health care workers on processes involved in performance management. In addition, the health service managers should ensure that the individual performance of health care workers is aligned to the mission and objectives of the health sector.

### Performance management planning

The health service managers should establish and agree upon the performance expectations of the workers. They need to ensure that all health care workers are clear on benchmarks they will be evaluated upon. Performance plans should be made for each individual worker and should be agreed and signed by both parties.

### Performance review

The performance assessments must be based on targets that were agreed upon during the performance planning meetings. The health service managers should use this performance planning meetings as an opportunity to identify the development needs of their workers. There has to be on-going feedback and coaching throughout the performance cycle.

### Performance feedback

Ongoing two-way communication should be encouraged throughout the performance cycle. The health service managers must provide regular and constructive feedback to the health care workers regarding their performance. This feedback should be properly documented for future reference. In addition to the feedback the health care workers receive from their managers they should also get feedback from other sources such as the clients and suppliers.

### Coaching

The health service managers should focus on improving their current performance and support the health care workers to build their capabilities for the future.

### Staff training and development

The health service managers must institute mechanisms to keep health care workers abreast with up-to-date knowledge and skills in their profession. There should also be equity in the management and administration of training opportunities at both local and national government levels.

### Rewards and recognition

The health service managers need to create links between performance appraisal results and the reward system in the districts. The rewards and recognition system should be devolved uniformly right from the National MoH to the health facility levels.

## Limitations of the study

The study focused on three categories of health care workers: doctors, clinical officers and professional nurses/midwives. The other categories of health care workers such as laboratory, pharmacy and dental technologists/assistants might have had different views about performance management which have not been captured by this study. In addition, the study was conducted in only four out of 112 districts in Uganda. These four districts were all located in the eastern region of the country; therefore, the results may not be generalised to the entire country. The semi-structured interviews were conducted with 21 health service managers who agreed to participate. It cannot be assumed that their ideas about the performance of health workers are the same as those managers who were not interviewed.

## Recommendations for further research

Based on the results of the study, it is recommended that qualitative research should also be carried out among health workers to obtain in-depth information about the factors that enhance or impede their performance. Other regions and districts of Uganda should also be included in the research. Research should be conducted to establish the effects of feedback given to health care workers by managers on whether and how this feedback changes their performance behaviour.

## Conclusions

Although there is a performance management system developed by the Ministry of Public Service the research uncovered a number of loopholes in its implementation in the health sector. In many instances no target setting and planning for performance management were done. The indicators for measuring performance were not clearly defined to the individuals to be assessed, and to the managers. Performance assessments were irregular, and not done at all in some districts. Career progression possibilities were limited and staff training and development seemed to be lacking in most districts. Additionally, performance feedback mechanisms appeared not to be functioning as well as expected at all levels of service delivery. The findings of this study have implications for all the stakeholders involved in the management of the human resources for health at national, district and health facility levels. The ministries of health, local governments and public service, as well as the bilateral and multilateral partners, need to discuss the issues and adopt the recommendations raised by this study. If this is done, it is hoped that key issues that are brought forward by this research will be used as a basis for improving the performance management of health care workers. This will, in turn, improve the outputs related to the current Ugandan national health sector strategic plan.

## Abbreviations

HC: Health centre; HMIS: Health management information system; MoH: Ministry of health; VHTs: Village health teams; WHO: World Health Organization.

## Competing interests

The authors declare that they have no competing interests.

## Authors’ contributions

GWL carried out this research for his Doctoral thesis; JHR and BLD were the promoters. GWL requested and obtained permission for the research, collected, analysed and interpreted the data. JHR and BLD provided the overall guidance of this research right from its inception to the end. GWL wrote the first draft of the article. Both JHR and BLD critically reviewed and contributed significantly to the article. GWL, JHR and BLD read and approved the final manuscript.

## Pre-publication history

The pre-publication history for this paper can be accessed here:

http://www.biomedcentral.com/1472-6963/13/355/prepub
